# Comparison of Cariogenic Organic Acid Concentrations According to Combined Use of Sucrose and Sugar Alcohols

**DOI:** 10.3390/jcm14217535

**Published:** 2025-10-24

**Authors:** Su-Yeon Hwang, Jung-Eun Park

**Affiliations:** 1 Department of Dental Hygiene, Daejeon Institute of Science and Technology, Daejeon 35408, Republic of Korea; hsyen@dst.ac.kr; 2 Department of Dental Hygiene, College of Health Science, Dankook University, Cheonan 31116, Republic of Korea

**Keywords:** dental caries, HPLC, ion chromatography, organic acid, saliva

## Abstract

**Objective:** This study involves a quantitative analysis of organic acids (lactate, acetate, propionate, formate, butylate, pyruvate, and valeric acid) concentrations after applying sucrose and sugar alcohols such as xylitol and erythritol in the oral cavity. **Methods:** Saliva samples were obtained from five volunteers before and up to 60 min after oral washing with sucrose and sugar alcohol (xylitol and erythritol). Concentrations of seven organic acids (lactate, acetate, propionate, formate, butyrate, pyruvate, and valerate) were determined by high-performance liquid chromatography using ion chromatography with conductivity detection. **Results:** The combination of sucrose and sugar alcohols, particularly erythritol, led to lower average lactate levels compared with 10% sucrose. Moreover, 1:1 post hoc analysis revealed that the levels of acetate and propionate decreased in the group treated with 2.5% and 7.5% of erythritol compared with those treated with xylitol (*p* < 0.05). However, the results did not indicate concentration dependence; owing to the small sample size (*n* = 5) these findings must be interpreted with care. This necessitates follow-up research, including oral bacterial testing and studies with larger sample sizes. **Conclusions:** The concentrations of organic acids varied with respect to the combined administration of sucrose and sugar alcohols. Further studies are necessary to investigate the effect of other sugar alcohols on bacterial growth.

## 1. Introduction

Dental caries is estimated to affect more than one-third of the global population and is the most prevalent oral condition persisting as a major public health issue [1,2]. It is caused by demineralization of the dental hard tissues caused by a variety of organic acids formed in the process of carbohydrate fermentation by acid-forming bacteria in dental biofilms [3].

Dental caries is a multifactorial disease that is caused by a combination of factors, including socioeconomic, genetic, and behavioral factors, oral pH, microorganisms, and dietary plans [4,5]. The WHO has implicated the intake of free sugars to be the principal cause of dental caries [6]. An earlier systematic review on the amount of sugars and the risk of caries revealed that limiting free sugar intake to less than 10% of total energy intake corresponds to a lower risk of dental caries [7]. Therefore, reducing sugar consumption and using sugar substitutes are suggested to reduce the daily sugar intake.

Erythritol and xylitol are widely employed as sugar substitutes; these contain molecular structures with aldehydes and ketones reduced to alcohols and all oxygen atoms present in the form of hydroxyl groups [8,9]. The preventive and inhibitory efficacy of sugar alcohols has been acknowledged in both scientific literature reviews and commercial product formulations. The identified anti-caries mechanisms of these components include reduction in cariogenic biofilm development, growth inhibition of caries inducing bacteria, and reduction in the expression levels of sucrose-metabolizing genes [10,11,12].

The cariogenic potential of erythritol and xylitol has been underexplored, whereas considerable research focus has been placed on the investigation of oral bacteria [13,14]. In addition, studies on the preventive efficacy of xylitol on dental caries report limitations in terms of the appropriateness of research methodology and potential bias. In earlier studies without the control group, the amount of saliva increased by mechanical action was not controlled. Moreover, as the studies relied solely on the count of *S. mutans*, the role of *Lactobacillus* spp. and *Actinomyces*, which could also cause dental caries, were not considered [15]. Despite substantial prior investigation, the degree of caries-inducing organic acid production remains undetermined. Furthermore, the inhibitory effect of sugar alcohols on reducing the organic acids produced by sucrose requires comparative assessment.

In this study, the changes in the concentrations of organic acids produced by oral bacteria after the combined use of xylitol and erythritol with sucrose have been quantified. Using this approach, their cariogenic potential is assessed while addressing the limitations of previous studies. Since the organic acids, which are biochemical metabolites, are quantified and assessed, rather than simply measuring bacterial counts, this approach provides deeper insights into the actual dietary and oral environment.

In this study, 10% sucrose mixed with the different concentrations of xylitol and erythritol were applied in the oral cavity. Then, the levels of the six major organic acids (lactate, acetate, propionate, formate, butylate, pyruvate, and valeric acid) formed in saliva after the treatment were quantitatively analyzed using high-performance liquid chromatography (HPLC) using ion chromatography with conductivity detection (IC-CD). By comparing the effects of xylitol and erythritol on cariogenic organic acids produced from sucrose, the dietary regulation approaches to prevent dental caries are analyzed.

## 2. Materials and Methods

### 2.1. Reagents and Solutions

Carboxylic acid standards were obtained from Sigma Chemical Co. (St. Louis, MO, USA). Sodium fluoride standard was obtained from Dionex (Sunnyvale, CA, USA). All aqueous solutions were prepared using high-purity water (18 MΩ resistivity) obtained with a Milli-Q system (Millipore, Bedford, MA, USA). HPLC (IC-CD)-grade sodium hydroxide (NaOH) was purchased from Fisher Scientific (Fairlawn, NJ, USA). All other reagents and solvents were of guaranteed or analytical grade.

A Millipore membrane filter (type HA, pore size = 0.45 mm, with mixed cellulose ester composition) was used for solvent filtration. All samples were filtered before they were injected into an ion chromatography apparatus (Dionex) through disposable syringe filters [hydrophobic polytetrafluoroethylene (PTFE) membrane, with pore size = 0.2 µm], which were obtained from Advantec MFS, Inc. (Tokyo, Japan).

### 2.2. Saliva Sample Preparation

This study complied with the guidelines of the Declaration of Helsinki and ethical approval for this study was obtained from the institutional review board of Dankook University (DKU-2022-02-002-002). This study involved five healthy Korean volunteers. Those with dental caries, implants, diabetes, gingival inflammation, or metabolic disease and who were current smokers were excluded. All volunteers were female, with an average age of 28.6 years. Volunteers were asked to refrain from eating, drinking, brushing their teeth, or gargling for three hours before saliva collection. Unstimulated fasting samples were collected as control before sugar treatment. Then, the volunteers rinsed their mouths with 25 mL of a mixture of sugar and sugar alcohol and provided saliva samples after 0, 1, 5, 10, 30 and 60 min after rinsing. A total of 11 types of sugar mixtures were applied to participants including sucrose 10% (A), sucrose 10% and erythritol 1, 2.5, 5, 7.5, and 10% (B), and sucrose 10% and xylitol 1, 2.5, 5, 7.5, and 10% (C). Each volunteer participated in a total of 11 experiments for two months under the same condition. A three-day washout interval was included between the oral wash periods of each test solution. Collected saliva samples were immediately filtered through a PTFE membrane (pore size = 0.2 µm) in a disposable syringe filter to separate out enzymes and bacteria, divided into ten sterilized tubes and kept frozen until use. To analyze the organic acid content, frozen saliva samples were thawed and diluted 20-fold with deionized water.

### 2.3. HPLC (IC-CD)

Organic acids and sodium fluoride were analyzed using a Dionex ion chromatography system comprising an ED50 conductivity detector, and an LC20 chromatography enclosure; with a 10 mL sample loop; and suppressor operated in the auto suppression recycle mode. The suppressor current was 300 mA. Separation was performed on a Dionex IonPac AS11-HC (250 mm × 4 mm) equipped with an Ion Pac AG11-HC column (50 mm × 4 mm) guard column. The procedure involved an isocratic elution of A:B (98.6:1.4) for 20 min. The compounds were analyzed at room temperature, and the flow rate of the mobile phase was 1 mL/min. Solvent composition was optimized by appropriately modifying the conditions used in previous studies [3]. Data acquisition and instrument control were performed by using the Dionex Chromeleon program (6.8 version). The mobile phase was created by degassing with vacuum filtration, followed by sonication for 20 min before use. The NaOH solution was purged with helium throughout the experiment to remove carbonate from water.

### 2.4. Method Validation

The linearity of the detection response for each compound was analyzed by using seven carboxylic acid standards (0.01, 0.05, 0.1, 0.25, 0.5, 1, and 5 mM). The linear regression equation was y = ax + b, where y and x are the peak area and sample concentration, respectively. The limit of detection (LOD) and limit of quantification (LOQ) were determined according to ICH guideline Q2B. This study was carried out under the same experimental conditions of previous studies, which were comprehensively validated for accuracy and precision [3].

### 2.5. Statistical Analysis

Sample data were gathered from five subjects. Owing to the small sample size, applying the normal approximation using the central limit theorem is challenging. Hence, a non-parametric test was used to analyze the data. The Kruskal–Wallis test was used to test differences between groups with respect to sugar oral rinse. Moreover, the Bonferroni test was used as a post hoc test to compare all the combinations of pairs. Abnormal values in the dataset were eliminated. All statistical analyses were performed using Python ver. 3.7 software. Statistical significance was considered at *p* < 0.05, *p* < 0.01, and *p* < 0.001.

## 3. Results

### 3.1. Analysis of the Organic Acid in Saliva Samples Using HPLC (IC-CD)

**Figure 1:**
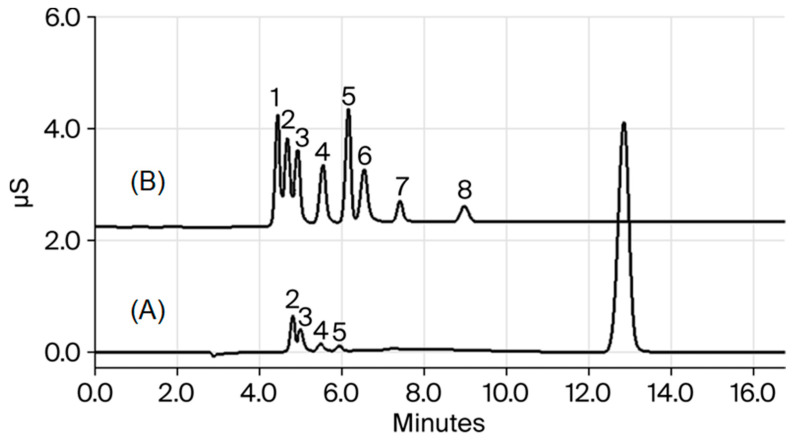
Chromatogram of the standard (A) and the saliva sample (B). 1. NaF; 2. lactate; 3. acetate; 4. propionate; 5. formate; 6. butyrate; 7. pyruvate; and 8. Valerate.

Table 1 presents the linear range, linear equation, correlation coefficient, LOD and LOQ of organic acids analyzed according to the HPLC (IC-CD) conditions. Figure 1 shows the chromatograms of the standard. The linear regression coefficients were 0.9988–0.9999 in the linear range of the chromatogram. The LODs were calculated as 0.37–1.32 µM in accordance with ICH guideline Q2B, and the LOQs were calculated to 1.12–3.95 µM. All compounds have good linearity (r^2^ > 0.99) of up to 50 µg/mL.

### 3.2. Organic Acid Levels in Saliva After Applying Sugar and Sugar Alcohols

Organic acid levels in the mouth increased immediately (0 min) after applying sugar and sugar alcohols. Concentrations of most organic acids decreased after 30 to 60 min after applying sugar and sugar alcohols. The measured levels of butyrate in most sugar alcohol groups and valerate in all groups were < 0.1. Contrastingly, no difference was observed in organic acid concentrations in the group with 10% sucrose + xylitol. (The detailed data are provided in the Appendix A).

### 3.3. Amount of Increase/Decrease in Organic Acids in Saliva After Applying Sugar and Sugar Alcohols

**Figure 2:**
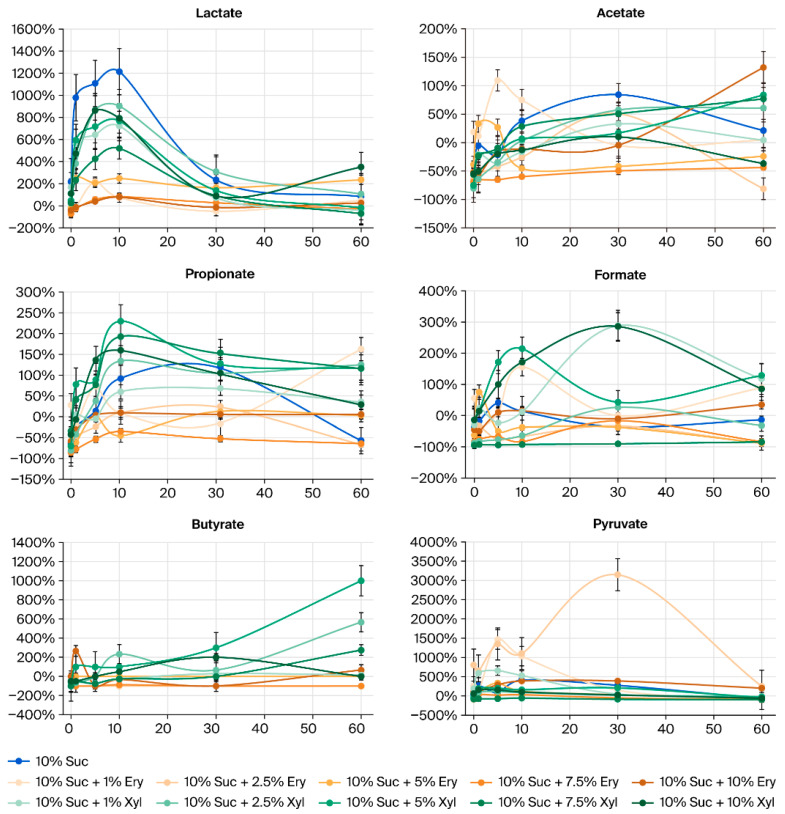
Amount of increase/decrease in organic acids in saliva after applying sugar and sugar alcohols.

**Figure 3:**
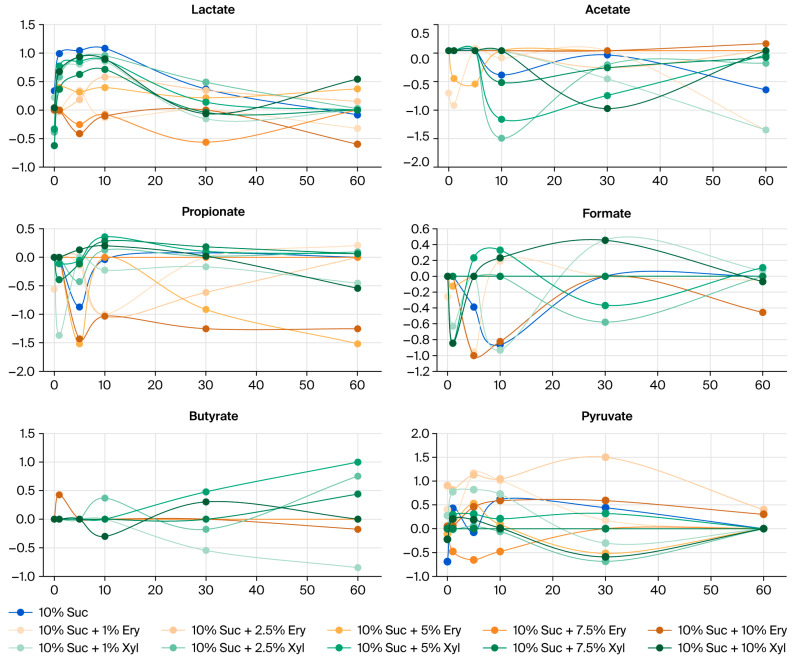
Log_10_-transformed percentage changes in salivary organic acids after applying sugar and sugar alcohols.

Figure 2 shows the degree of increase/decrease (%) in organic acid concentrations in saliva after applying sugar and sugar alcohols. And Figure 3 shows the Log_10_-transformed percentage changes in salivary organic acids after applying sugar and sugar alcohols. The organic acids lactate and acetate substantially increased in concentration after applying sugar. The average level of lactate, in particular, decreased after the combined administration of sugar and sugar alcohols compared to the control group treated with only 10% sucrose.

The erythritol group had a lower lactic acid concentration than the xylitol group. However, owing to the limited sample size, these findings must be interpreted with care. Initially, sugar alcohols had no effect on the level of acetate; however, the organic acid levels gradually decreased from 30 min after the treatment.

### 3.4. Comparison of Organic Acid Levels in the Erythritol Group and Other Groups (Sucrose and Xylitol)

The non-parametric test revealed a difference in organic acid concentrations. Table 2 represents the statistical significance assessed by comparing 10% sucrose + erythritol and other sugar alcohols using 1:1 post hoc analysis. Compared with the xylitol group, the erythritol group exhibited a distinct reduction in the levels of acetate and propionate at concentrations of 2.5% and 7.5%. A significant decrease was observed in the levels of formate at concentrations of 2.5%, 5%, and 7.5% erythritol.

## 4. Discussion

In this study, the concentrations of organic acids produced by pathogenic bacteria were quantitatively analyzed after applying a mixture of sucrose and sugar alcohols, such as erythritol and xylitol, in the oral cavity. The seven types of organic acids (lactate, acetate, propionate, formate, butylate, pyruvate, and valeric acid) are carboxylic acids, which are produced by microorganisms during the fermentation process of carbohydrates [3]. This research isolated seven different organic acids within 15 min using HPLC (IC-CD) analysis and sample pre-treatment. Moreover, all organic acids had a linearity greater than 0.998 in the linear range 0.01–5 mM, validating the analytical techniques.

The analysis results showed that butyrate in several sugar alcohol groups and valerate in all groups were < LOQ. In a previous study on the correlation between organic acids and negative ions after sugar consumption, butyrate and valerate were not detected in many participants [3]. This is because butyrate and valerate are volatile short chain fatty acids [16,17] produced by anaerobic bacteria such as *Clostridium* spp. [18]. Even though these acids cause halitosis, they are produced in small amounts during fermentation and are more frequently detected in the colon and feces [19]. Therefore, the detection of butyrate and valerate in small amounts aligned with the results of previous studies [3]. In this study, the mean concentrations of organic acids corresponding to different types and concentration of carbohydrates were compared. A significant difference was observed in the mean concentrations of organic acids (Appendix A, Figure 2). Lactate is the most common metabolic byproduct associated with the formation of dental caries. Lactate is known to accelerate demineralization of dental enamel faster than acetate or propionate [20,21]. Considering the aqueous systems of lactate, concentrations appear to be affected by soluble compounds in the saliva [22]. In this research, no statistical significance was detected in changes in lactate levels in each sugar and sugar alcohol group. However, a prominent decrease was observed in lactate concentration of the erythritol group than in the 10% sucrose group, as shown in the graph.

Furthermore, differences in the mean levels of formate were evident at 0, 5, 10, and 60 min; however, a corresponding regular pattern in the increase or decrease in formate levels could not be established. Formate is produced through degradation of lactate and acetate by oral bacteria (*Actinomyces* and *Fusobacterium*) [23,24]. A previous study on the antibacterial activity of fatty acids against oral microorganisms reported that formate showed an extensive inhibitory effect on oral bacteria [25]. Fatty acids released by bacteria have a significant role in oral microbial ecology and dental biofilm by affecting the growth of competitive microorganisms [25]. Similarly, different oral organic acids influenced each other, interacting with the oral environment and bacteria.

The relatively small sample size (five participants) limited the ability of detection of differences corresponding to multiple conditions involving different carbohydrates and concentrations. Although each condition was verified with 1:1 post hoc analysis, a reduction in organic acid levels dependent on increasing concentration was not found in the erythritol group. On the contrary, acetate and propionate exhibited a distinct decrease in the groups of 2.5% and 7.5% erythritol compared to xylitol. In the erythritol group, formate level decreased and the level of butyrate, which was mostly below detection limits, slightly decreased (*p* < 0.05) compared with the sucrose group (*p* < 0.05). Formate levels significantly decreased in the groups with erythritol at 2.5%, 5%, and 7.5% concentrations compared to the group containing only sucrose.

The relationship between sugar alcohols and dental caries has been extensively explored by prior research to explore the effect of erythritol, a polyol of the tetritol type, and xylitol on oral bacteria [12,26,27]. In a study on erythritol concentrations, erythritol at low concentrations (0.5–2%) exhibited a weaker inhibitory effect on bacterial growth, whereas erythritol at high concentrations (8–16%) strongly inhibited the acid formation of *S. mutans*.

Furthermore, 10% erythritol exhibited an inhibitory effect on the microstructure and metabolic profiles of dental biofilm composed of *P. gingivalis* and other bacteria [28]. This study assessed organic acids in relatively high sugar alcohol concentrations (higher than 10% compared to sucrose). Therefore, further investigation is required to assess the effect of applying sugar alcohols at an appropriate range of concentrations on oral bacteria. Low levels of acetate and propionate indicated that erythritol was more effective than xylitol at inhibiting production of organic acids that induce dental caries. Previous research focused on the microbial community and metabolites such as lactate, acetate, and pyruvate in dental caries, particularly their relationship with biofilms [29]. In particular, acetic acid is produced by anaerobic bacteria playing a significant role in carbohydrate and lipid metabolism [30]. Therefore, the formation of organic acids can be affected by bacterial species and the type of sugars inducing dental caries.

Preliminary research indicated that the erythritol group had lower dental biofilm weight and less tooth surfaces developing into enamel or dentin caries compared to the xylitol group [26,27]. A long-term comparative study on dental caries provided strong evidence that erythritol was more effective in preventing dental caries than xylitol. The mechanism of inhibitory effect of erythritol is related to reduction in adherence of oral bacteria. Erythritol was found to reduce counts of bacteria by suppressing adherence of *S. mutans*, *S. sanguinis*, and other bacterial pathogens residing in the oral cavity [31]. Furthermore, previous research developed a compound consisting of erythritol and zinc chloride and evaluated its penetration and bactericidal activity against *S. mutans* biofilms. The erythritol and zinc chloride complex reduced the interaction between bacterial polysaccharides, enabling its penetration into the biofilm to inhibit and reduce the bacteria [32]. In addition, bacterial growth can be suppressed using different approaches such as DNA and RNA depletion, acquisition of peptides, change in amino acid metabolism, and reduction in extracellular matrix formation [33]. These findings indicate that erythritol reduces oral bacteria associated with caries development. In our study, acetate, propionate and other organic acids were detected at low concentrations in the erythritol group compared to the xylitol group. Previous studies indicate that erythritol reduces the amounts of organic acids, the metabolic byproducts with its inhibitory effect of bacterial growth.

Dental caries develops over a long time owing to multiple factors including host saliva and oral hygiene. Dental caries has been extensively investigated. Since the primary cause of dental caries is organic acids in the oral cavity, a quantitative analysis of organic acids in this study is appropriate to determine the factors of dental caries in a short period of time. Moreover, ion chromatographic determination of organic acids is the preferred analytical method for observing the formation and suppression of organic acids, the causative substances of dental caries.

The limitations of this study are as follows: first, the limited sample size (*n* = 5) may affect the average values of individual salivary components and organic acid concentrations. Hence, these results cannot be generalized and should be interpreted with caution. Second, the habits of the volunteers, compliance, and oral condition changes during the repeated oral wash could have influenced the results. Moreover, the fixed application order of the solution reduces randomization. Third, the washout period was set to three days. Therefore some changes in the oral environment could not have reached the baseline, affecting the next saliva sample. Therefore, further research with increased sample size, randomized order and sufficient washout period is necessary. This study offers the advantage of quantifying organic acid concentrations through direct biochemical analysis, rather than simply measuring the bacterial count, despite its small sample size. Furthermore, validating the HPLC (IC-CD) analysis method ensured the reliability and scientific basis of the actual salivary organic acid concentration data in the oral cavity. In addition, the distribution of different organic acids with respect to the concentration sucrose, xylitol, and erythritol and time period was established.

In contrast to previous conventional studies, this study was meaningful in that it modified comparative studies on oral bacteria through the comparison of organic acid concentrations by applying erythritol and xylitol, gaining more popularity. This quantitative analysis of organic acids can be expanded by including the analysis of more numbers of bacterial species.

## 5. Conclusions

In this study, the average levels of lactate were observed to be reduced in the groups treated with a combination of sucrose and sugar alcohols (in particular, erythritol) compared with the group with 10% sucrose alone after applying sucrose and sugar alcohols. Moreover, 1:1 post hoc analysis revealed that the levels of acetate and propionate decreased in the group treated with 2.5% and 7.5% of erythritol compared to xylitol (*p* < 0.05). However, the dependence on concentration levels was not observed, which should be interpreted with caution given the small sample size (*n* = 5). Hence, follow-up research is needed, including oral bacterial testing and studies with larger sample sizes.

## Figures and Tables

**Table 1:** Linear ranges, linear equations, and coefficients of the organic acid.

Compounds	Linear Range (mM)	Linear Equation	r^2^	LOD (µM)	LOQ (µM)
Lactate	0.01–5	y = 0.3481x + 7.6541	0.9988	0.62	1.84
Acetate	0.01–5	y = 0.5411x + 15.29	0.9998	1.05	3.18
Propionate	0.01–5	y = 0.272x + 5.9467	0.9999	1.32	3.95
Formate	0.01–5	y = 0.4988x + 7.1799	0.9995	0.58	1.76
Butyrate	0.01–5	y = 0.3084x + 3.3577	0.9995	0.53	1.61
Pyruvate	0.01–5	y = 0.1361x + 1.53	0.9998	0.37	1.12
Valerate	0.01–5	y = 0.1701x + 2.1809	0.9999	0.51	1.56

LOD, limit of detection; LOQ, limit of quantitation.

**Table 2:** One-to-one comparison between sugar/sugar alcohol groups (Bonferroni post hoc analysis).

		Lactate			Acetate			Propionate			Formate			Butyrate			Pyruvate		
		Statistic	*p*	*p*_corr	Statistic	*p*	*p*_corr	Statistic	*p*	*p*_corr	Statistic	*p*	*p*_corr	Statistic	*p*	*p*_corr	Statistic	*p*	*p*_corr
10% Suc + 1% Ery	S1	−0.429	0.669	1	−1.988	0.051	1	−1.38	0.172	1	−2	0.05	1	−1.406	0.164	1	−2.4	0.019	1
	E2.5	3.099	0.003	0.155	2.276	0.026	1	2.634	0.01	0.573	2.706	0.009	0.473	−2.06	0.043	1	0.696	0.489	1
	E5	0.45	0.654	1	−0.212	0.833	1	0.168	0.867	1	2.157	0.035	1	−1.062	0.292	1	−0.741	0.461	1
	E7.5	3.102	0.003	0.154	1.908	0.061	1	2.195	0.032	1	2.643	0.01	0.561	−1.54	0.128	1	1.741	0.086	1
	E10	2.209	0.031	1	−0.135	0.893	1	0.413	0.681	1	−1.182	0.241	1	−2.337	0.022	1	0.548	0.586	1
	X1	0.011	0.991	1	−2.594	0.012	0.639	−3.298	0.002	0.085	−1.771	0.081	1	−3.843	0	0.014	−2.053	0.044	1
	X2.5	0.772	0.443	1	−1.696	0.094	1	−2.149	0.035	1	−2.419	0.018	1	−2.888	0.005	0.285	−0.22	0.826	1
	X5	0.171	0.865	1	−1.581	0.118	1	−1.508	0.136	1	0.003	0.998	1	−2.999	0.004	0.208	−1.923	0.059	1
	X7.5	−0.381	0.705	1	−3.171	0.002	0.125	−2.863	0.006	0.307	−1.244	0.218	1	−3.172	0.002	0.125	−1.819	0.073	1
	X10	1.24	0.219	1	−1.746	0.085	1	−0.879	0.383	1	−0.184	0.855	1	−3.908	0	0.011	−2.114	0.038	1
10% Suc + 2.5% Ery	S1	−2.867	0.006	0.302	−3.104	0.003	0.152	−2.512	0.014	0.791	−5.232	0	0	1.773	0.081	1	−2.711	0.009	0.467
	E5	−2.216	0.03	1	−2.029	0.046	1	−1.788	0.078	1	−0.491	0.625	1	−0.633	0.529	1	−1.344	0.183	1
	E7.5	−0.114	0.909	1	−0.584	0.561	1	−0.714	0.478	1	−0.263	0.794	1	−0.567	0.573	1	1.159	0.251	1
	X1	−2.528	0.014	0.758	−4.28	0	0.003	−5.52	0	0	−2.966	0.004	0.228	−3.191	0.002	0.118	−2.335	0.023	1
	X2.5	−1.69	0.096	1	−3.017	0.004	0.197	−3.324	0.001	0.078	−3.222	0.002	0.107	−2.509	0.015	0.796	−1.122	0.266	1
	X5	−2.195	0.032	1	−3.171	0.002	0.125	−3.478	0	0.048	−2.89	0.005	0.284	−2.19	0.032	1	−2.425	0.018	0.987
	X7.5	−2.777	0.007	0.389	−4.784	0	0	−4.513	0	0.001	−1.623	0.109	1	−2.537	0.014	0.741	−2.027	0.047	1
	X10	−1.546	0.127	1	−3.85	0	0.014	−3.799	0	0.017	−2.869	0.006	0.301	−2.23	0.029	1	−3.186	0.002	0.12
10% Suc + 5% Ery	S1	−0.79	0.433	1	−1.748	0.085	1	−1.399	0.166	1	−4.489	0	0.001	1.01	0.316	1	−1.934	0.057	1
	E7.5	2.195	0.032	1	1.705	0.093	1	1.445	0.153	1	0.319	0.751	1	0.332	0.741	1	2.145	0.036	1
	X1	−0.39	0.698	1	−2.13	0.037	1	−3.037	0.003	0.186	−2.78	0.007	0.386	−1.496	0.139	1	−1.633	0.107	1
	X2.5	0.319	0.751	1	−1.42	0.16	1	−2.126	0.037	1	−3.104	0.003	0.153	−1.551	0.126	1	0.65	0.518	1
	X5	−0.223	0.824	1	−1.249	0.216	1	−1.467	0.147	1	−2.277	0.026	1	−0.642	0.523	1	−1.224	0.225	1
	X7.5	−0.74	0.462	1	−2.677	0.009	0.511	−2.734	0.008	0.438	−1.572	0.121	1	−1.077	0.285	1	−1.507	0.136	1
	X10	0.699	0.487	1	−1.269	0.209	1	−0.883	0.38	1	−2.326	0.023	1	−0.198	0.844	1	−0.912	0.365	1
10% Suc + 7.5% Ery	S1	−2.853	0.006	0.315	−2.91	0.005	0.268	−2.325	0.023	1	−5.236	0	0	1.42	0.16	1	−3.068	0.003	0.17
	X1	−2.511	0.014	0.793	−4.066	0	0.007	−5.231	0	0	−2.924	0.005	0.257	−2.526	0.014	0.763	−2.656	0.01	0.54
	X2.5	−1.66	0.102	1	−2.794	0.007	0.371	−3.138	0.003	0.138	−3.192	0.002	0.117	−2.166	0.034	1	−2.791	0.007	0.374
	X5	−2.172	0.033	1	−2.923	0.005	0.258	−3.178	0.002	0.122	−2.833	0.006	0.333	−1.475	0.145	1	−3.042	0.003	0.183
	X7.5	−2.761	0.007	0.407	−4.589	0	0.001	−4.275	0	0.003	−1.605	0.113	1	−1.939	0.057	1	−2.261	0.027	1
	X10	−1.513	0.135	1	−3.602	6E−04	0.032	−3.369	0.001	0.068	−2.81	0.007	0.355	−1.027	0.308	1	−4.987	0	0
10% Suc + 10% Ery	S1	−2.221	0.03	1	−1.89	0.063	1	−1.602	0.114	1	−0.371	0.712	1	2.273	0.026	1	−2.658	0.01	0.538
	E2.5	0.898	0.373	1	2.284	0.026	1	2.915	0.005	0.264	3.196	0.002	0.116	1.791	0.078	1	0.195	0.846	1
	E5	−1.493	0.14	1	−0.093	0.926	1	−0.13	0.897	1	2.835	0.006	0.331	0.693	0.491	1	−1.236	0.221	1
	E7.5	0.842	0.403	1	1.931	0.058	1	2.352	0.022	1	3.144	0.003	0.135	1.328	0.189	1	1.486	0.142	1
	xy1	−1.849	0.069	1	−2.418	0.018	1	−3.901	0	0.012	−0.927	0.357	1	−0.859	0.393	1	−2.284	0.026	1
	xy2.5	−1.041	0.302	1	−1.581	0.119	1	−2.401	0.019	1	−1.811	0.075	1	−1.037	0.303	1	−0.958	0.342	1
	xy5	−1.576	0.12	1	−1.441	0.154	1	−1.934	0.057	1	1.212	0.23	1	0.151	0.881	1	−2.349	0.022	1
	xy7.5	−2.145	0.036	1	−2.991	0.004	0.212	−3.267	0.002	0.093	−0.961	0.34	1	−0.394	0.695	1	−1.987	0.051	1
	xy10	−0.781	0.438	1	−1.548	0.126	1	−1.466	0.147	1	1.029	0.307	1	0.773	0.442	1	−3.098	0.003	0.156

## Data Availability

The original contributions presented in the study are included in the article, further inquiries can be directed to the corresponding authors.

## Appendix A

**Table A1:** Levels of organic acid produced after applying sugar and sugar alcohols at different concentrations mM (mean ± sd).

Variables	Min	Lactate	Acetate	Propionate	Formate	Butyrate	Pyruvate	Valerate
10% Sucrose	Control	0.68 ± 0.63	3.06 ± 5.38	0.82 ± 1.62	0.22 ± 0.2	<LOQ	0.44 ± 0.88	<LOQ
	0	2.16 ± 1.75	1.47 ± 1.32	0.29 ± 0.28	0.14 ± 0.09	<LOQ	0.53 ± 1.08	<LOQ
	1	7.33 ± 4.75	2.89 ± 2.69	0.62 ± 0.48	0.18 ± 0.19	<LOQ	1.61 ± 1.61	<LOQ
	5	8.21 ± 7.15	2.4 ± 2.62	0.93 ± 1.45	0.31 ± 0.2	<LOQ	0.81 ± 2.44	<LOQ
	10	8.93 ± 3.29	4.23 ± 8.78	1.57 ± 3.38	0.25 ± 0.11	<LOQ	2.25 ± 3.39	<LOQ
	30	2.27 ± 4.81	5.64 ± 1.25	1.8 ± 0.67	0.14 ± 0.24	<LOQ	1.65 ± 0.32	<LOQ
	60	1.24 ± 1.67	3.71 ± 4.09	0.35 ± 0.2	0.19 ± 0.12	<LOQ	0.14 ± 0.16	<LOQ
10% Suc + 1% Ery	Control	2.99 ± 3.14	1.39 ± 2.38	0.4 ± 0.59	0.09 ± 0.15	<LOQ	0.06 ± 0.08	<LOQ
	0	1.42 ± 0.97	1.65 ± 0.84	0.51 ± 0.52	0.14 ± 0.17	<LOQ	0.21 ± 0.28	<LOQ
	1	2.63 ± 3.27	1.55 ± 1.57	0.33 ± 0.3	0.06 ± 0.07	<LOQ	0.13 ± 0.25	<LOQ
	5	9.53 ± 2.81	2.91 ± 1.22	0.69 ± 0.3	0.1 ± 0.1	<LOQ	0.94 ± 0.56	<LOQ
	10	5.19 ± 1.45	2.43 ± 1.29	0.44 ± 0.33	0.23 ± 0.09 *	<LOQ	0.68 ± 0.25	<LOQ
	30	1.52 ± 3.38	1.32 ± 2.24	0.33 ± 0.63	0.09 ± 0.2	<LOQ	0.15 ± 1.05	<LOQ
	60	4.42 ± 2.9	1.45 ± 1.48 *	1.05 ± 0.87	0.17 ± 0.28	<LOQ	<LOQ	<LOQ
10% Suc + 2.5% Ery	Control	0.77 ± 3.79	1.44 ± 2.06	0.33 ± 0.45	0.09 ± 0.34	<LOQ	0.02 ± 0.18	<LOQ
	0	0.63 ± 1.06	0.51 ± 0.62	0.14 ± 0.21	0.05 ± 0.2	0.02 ± 0.07	0.18 ± 0.26	<LOQ
	1	1.49 ± 1.94	0.65 ± 0.54	0.18 ± 0.36	0.06 ± 0.1	0.01 ± 0.21	0.15 ± 0.23	<LOQ
	5	1.95 ± 2.75	0.83 ± 1.24	0.25 ± 0.28	0.02 ± 0.37	0 ± 0.03	0.29 ± 0.4	<LOQ
	10	3.7 ± 1.43	1.07 ± 1.19	0.36 ± 0.32	0.03 ± 0.22 *	<LOQ	0.24 ± 0.63	<LOQ
	30	2.48 ± 3.54	2.17 ± 1.45	0.41 ± 0.58	0.06 ± 0.3	<LOQ	0.65 ± 0.62	<LOQ
	60	1.86 ± 2.75	0.27 ± 2.5 *	0.11 ± 0.3	0.01 ± 0.23	0.02 ± 0.07	0.07 ± 0.31	<LOQ
10% Suc + 5% Ery	Control	1.39 ± 0.55	2.2 ± 2.38	0.66 ± 0.56	0.08 ± 0.15	0.12 ± 0.00	0.23 ± 0.04	<LOQ
	0	1.09 ± 0.37	1.35 ± 0.29	0.18 ± 0.07	0.03 ± 0.03	0.01 ± 0.04	0.41 ± 0.19	<LOQ
	1	4.87 ± 1.53	2.93 ± 0.54	0.25 ± 0.17	0.14 ± 0.06	0 ± 0.01	0.5 ± 0.12	<LOQ
	5	4.26 ± 2.99	2.79 ± 1.35	0.68 ± 0.37	0.04 ± 0.04	0 ± 0.01	1.00 ± 0.3	<LOQ
	10	4.83 ± 1.49	1.2 ± 2.32	0.36 ± 0.36	0.05 ± 0.08	<LOQ	0.51 ± 0.92	<LOQ
	30	3.67 ± 1.54	1.28 ± 0.79	0.74 ± 0.25	0.05 ± 0.01	0 ± 0.01	0.3 ± 0.4	<LOQ
	60	4.66 ± 1.87	1.67 ± 0.26 *	0.68 ± 0.06	0.01 ± 0.01	0 ± 0.04	0.06 ± 0.11	<LOQ
10% Suc + 7.5% Ery	Control	1.79 ± 1.82	2.37 ± 2.33	0.65 ± 0.88	0.12 ± 0.1	0.08 ± 0.28	0.18 ± 0.51	<LOQ
	0	0.47 ± 0.7	0.73 ± 1.33	0.09 ± 0.12	0.02 ± 0.05	0 ± 0.02	0.02 ± 0.35	<LOQ
	1	1.32 ± 4.84	0.82 ± 2.77	0.14 ± 0.26	0.03 ± 0.21	<LOQ	0.24 ± 0.6	<LOQ
	5	2.79 ± 5.19	0.82 ± 1.46	0.3 ± 0.49	0.04 ± 0.09	<LOQ	0.22 ± 0.72	<LOQ
	10	3.28 ± 3.88	0.95 ± 1.23	0.42 ± 0.94	0.02 ± 0.1 *	0.01 ± 0.00	0.24 ± 0.49	<LOQ
	30	2.28 ± 6.1	1.19 ± 4.01	0.31 ± 1.1	0.1 ± 0.09	<LOQ	0.09 ± 1.73	<LOQ
	60	1.26 ± 4.57	1.33 ± 2.48 *	0.23 ± 1.24	0.02 ± 0.02	<LOQ	<LOQ	<LOQ
10% Suc + 10% Ery	Control	2.1 ± 1.44	1.9 ± 1.68	0.54 ± 0.44	0.2 ± 0.07	0.03 ± 0.11	0.08 ± 0.16	<LOQ
	0	1.2 ± 0.52	0.91 ± 0.6	0.22 ± 0.08	0.11 ± 0.02	0.03 ± 0.00	0.17 ± 0.05	<LOQ
	1	1.81 ± 0.91	0.79 ± 0.83	0.37 ± 0.04	0.1 ± 0.02	0.11 ± 0.00	0.17 ± 0.22	<LOQ
	5	2.91 ± 1.9	1.6 ± 0.53	0.56 ± 0.26	0.22 ± 0.03	<LOQ	0.31 ± 0.26	<LOQ
	10	3.75 ± 1.96	1.68 ± 0.98	0.59 ± 0.2	0.23 ± 0.07 *	0.02 ± 0.00	0.39 ± 0.12	<LOQ
	30	1.78 ± 1.75	1.81 ± 0.61	0.57 ± 0.2	0.18 ± 0.03	<LOQ	0.39 ± 0.22	<LOQ
	60	2.63 ± 1.01	4.41 ± 1.96 *	0.57 ± 0.28	0.27 ± 0.04	0.05 ± 0.00	0.24 ± 0.00	<LOQ
10% Suc + 1% Xyl	Control	0.98 ± 0.81	3.68 ± 2.47	0.94 ± 0.43	0.17 ± 0.1	0.07 ± 0.07	0.26 ± 0.23	<LOQ
	0	2.62 ± 2.08	1.6 ± 1.34	0.55 ± 0.64	0.12 ± 0.11	0.03 ± 0.06	0.75 ± 0.67	<LOQ
	1	6.46 ± 3.83	2.92 ± 1.73	0.98 ± 0.53	0.21 ± 0.16	0.03 ± 0.04	1.8 ± 2.37	<LOQ
	5	7.27 ± 6.29	2.31 ± 1.99	0.89 ± 0.7	0.13 ± 0.1	0.02 ± 0.06	1.97 ± 2.84	<LOQ
	10	8.05 ± 1.38	3.07 ± 5.16	1.5 ± 1.64	0.19 ± 1.02	0.05 ± 0.14	1.64 ± 0.31	<LOQ
	30	1.67 ± 6.4	4.89 ± 1.95	1.58 ± 0.63	0.66 ± 0.08	0.09 ± 0.03	0.39 ± 3.44	<LOQ
	60	0.57 ± 0.25	3.84 ± 2.7	1.27 ± 0.87	0.37 ± 0.43	0.08 ± 0.12	0.18 ± 0.17	<LOQ
10% Suc + 2.5% Xyl	Control	0.68 ± 0.56	3.19 ± 3.74	0.8 ± 0.54	0.72 ± 0.05	0.03 ± 0.04	0.24 ± 0.41	<LOQ
	0	0.96 ± 0.42	0.62 ± 0.74	0.15 ± 0.14	0.09 ± 0.02	0.01 ± 0.01	0.19 ± 0.44	<LOQ
	1	3.28 ± 1.92	1.15 ± 0.83	0.46 ± 0.21	0.13 ± 0.03	0.01 ± 0.02	0.47 ± 0.7	<LOQ
	5	6.56 ± 5.33	2.08 ± 0.78	1.1 ± 0.47	0.17 ± 0.22	0.03 ± 0.03	0.51 ± 0.49	<LOQ
	10	6.81 ± 1.18	3.29 ± 2.78	1.87 ± 0.69	0.26 ± 0.29	0.1 ± 0.06	0.45 ± 0.35	<LOQ
	30	2.78 ± 3.68	5.02 ± 1.23	1.64 ± 0.54	0.91 ± 0.16	0.05 ± 0.03	0.29 ± 0.36	<LOQ
	60	1.4 ± 5.47	5.13 ± 1.49	1.8 ± 0.49	0.49 ± 0.18	0.2 ± 0.03	0.21 ± 0.1	<LOQ
10% Suc + 5% Xyl	Control	0.9 ± 0.53	2.71 ± 3.79	0.44 ± 1.18	0.07 ± 0.66	0.01 ± 0.07	0.36 ± 0.34	<LOQ
	0	1.32 ± 0.65	0.67 ± 0.51	0.13 ± 0.18	0.06 ± 0.05	0.00 ± 0.01	0.18 ± 0.21	<LOQ
	1	6.23 ± 2.51	2.1 ± 0.65	0.78 ± 0.31	0.08 ± 0.1	0.02 ± 0.02	1.06 ± 0.42	<LOQ
	5	7.34 ± 6.88	2.26 ± 2.58	0.83 ± 2.24	0.19 ± 0.2	0.02 ± 0.13	1.09 ± 0.39	<LOQ
	10	7.81 ± 3.2	2.89 ± 5.24	1.45 ± 1.76	0.22 ± 1.33	0.02 ± 0.08	0.94 ± 0.33	<LOQ
	30	2.14 ± 7.16	3.17 ± 1.95	1.00 ± 1.09	0.1 ± 0.21	0.04 ± 0.08	1.11 ± 0.62	<LOQ
	60	0.73 ± 1.8	4.98 ± 5.36	0.95 ± 2.2	0.16 ± 0.65	0.11 ± 0.26	0.21 ± 0.26	<LOQ
10% Suc + 7.5% Xyl	Control	1.59 ± 1.1	3.27 ± 2.22	0.64 ± 0.3	1.83 ± 0.07	0.04 ± 0.03	3.24 ± 0.43	<LOQ
	0	1.97 ± 0.62	1.43 ± 0.39	0.42 ± 0.09	0.1 ± 0.04	<LOQ	0.69 ± 0.17	<LOQ
	1	5.28 ± 4.98	2.49 ± 1.95	0.9 ± 0.8	0.14 ± 0.05	0.02 ± 0.03	0.86 ± 1.66	<LOQ
	5	8.32 ± 7.83	2.95 ± 1.78	1.13 ± 1.37	0.11 ± 0.27	0.01 ± 0.04	0.9 ± 1.00	<LOQ
	10	9.84 ± 2.38	4.2 ± 3.34	1.87 ± 1.14	0.14 ± 0.04	0.03 ± 0.05	1.38 ± 1.62	<LOQ
	30	3.04 ± 5.92	4.93 ± 1.09	1.62 ± 0.5	0.18 ± 0.21	0.04 ± 0.03	0.47 ± 1.41	<LOQ
	60	0.46 ± 0.54	5.78 ± 5.54	1.38 ± 0.97	0.29 ± 0.18	0.15 ± 0.14	0.15 ± 0.3	<LOQ
10% Suc + 10% Xyl	Control	0.6 ± 1.38	3.36 ± 3.11	0.42 ± 0.68	0.07 ± 3.28	0.02 ± 0.05	0.35 ± 6.56	<LOQ
	0	1.26 ± 1.15	1.51 ± 0.83	0.24 ± 0.32	0.06 ± 0.12	0.01 ± 0.00	0.56 ± 0.73	<LOQ
	1	3.43 ± 2.64	1.66 ± 1.7	0.39 ± 0.79	0.08 ± 0.17	0.01 ± 0.06	0.91 ± 0.7	<LOQ
	5	5.77 ± 7.71	2.68 ± 1.28	0.99 ± 1.21	0.14 ± 0.12	0.02 ± 0.04	0.89 ± 0.73	<LOQ
	10	5.35 ± 2.44	2.92 ± 3.8	1.09 ± 1.83	0.19 ± 0.18	0.03 ± 0.07	0.71 ± 0.34	<LOQ
	30	1.12 ± 6.72	3.7 ± 1.12	0.86 ± 0.66	0.27 ± 0.08	0.06 ± 0.02	0.44 ± 0.74	<LOQ
	60	2.7 ± 0.28	2.12 ± 4.77	0.54 ± 1.43	0.13 ± 0.34	0.02 ± 0.16	0.15 ± 0.15	<LOQ

LOQ, limit of quantitation. * *p* < 0.1, Difference in mean organic acid levels according to different concentrations of erythritol using Kruskal–Wallis test.
